# Doxycycline Leads to Sterility and Enhanced Killing of Female *Onchocerca volvulus* Worms in an Area With Persistent Microfilaridermia After Repeated Ivermectin Treatment: A Randomized, Placebo-Controlled, Double-Blind Trial

**DOI:** 10.1093/cid/civ363

**Published:** 2015-05-06

**Authors:** Alexander Yaw Debrah, Sabine Specht, Ute Klarmann-Schulz, Linda Batsa, Sabine Mand, Yeboah Marfo-Debrekyei, Rolf Fimmers, Bettina Dubben, Alexander Kwarteng, Mike Osei-Atweneboana, Daniel Boakye, Arcangelo Ricchiuto, Marcelle Büttner, Ohene Adjei, Charles D. Mackenzie, Achim Hoerauf

**Affiliations:** 1 Faculty of Allied Health Sciences, Kwame Nkrumah University of Science and Technology; 2 Kumasi Centre for Collaborative Research in Tropical Medicine, Ghana; 3 Institutes for Medical Microbiology, Immunology and Parasitology; 4 Medical Biometry, Informatics and Epidemiology, University of Bonn, Germany; 5 Council for Scientific and Industrial Research, Water Research Institute; 6 Noguchi Memorial Institute for Medical Research, University of Ghana, Accra; 7 Ahrensburger Stieg 17, Hamburg, Germany; 8 School of Medical Sciences, Kwame Nkrumah University of Science and Technology, Kumasi, Ghana; 9 Centre for Neglected Tropical Diseases, Liverpool School of Tropical Medicine, United Kingdom

**Keywords:** doxycycline, *Onchocerca volvulus*, suboptimal response, ivermectin, *Wolbachia*

## Abstract

***Background.*** Ivermectin (IVM) has been the drug of choice for the treatment of onchocerciasis. However, there have been reports of persistent microfilaridermia in individuals from an endemic area in Ghana after many rounds of IVM, raising concerns of suboptimal response or even the emergence of drug resistance. Because it is considered risky to continue relying only on IVM to combat this phenomenon, we assessed the effect of targeting the *Onchocerca volvulus Wolbachia* endosymbionts with doxycycline for these individuals with suboptimal response.

***Methods.*** One hundred sixty-seven patients, most of them with multiple rounds of IVM, were recruited in areas with IVM suboptimal response and treated with 100 mg/day doxycycline for 6 weeks. Three and 12 months after doxycycline treatment, patients took part in standard IVM treatment.

***Results.*** At 20 months after treatment, 80% of living female worms from the placebo group were *Wolbachia* positive, whereas only 5.1% in the doxycycline-treated group contained bacteria. Consistent with interruption of embryogenesis, none of the nodules removed from doxycycline-treated patients contained microfilariae, and 97% of those patients were without microfilaridermia, in contrast to placebo patients who remained at pretreatment levels (*P* < .001). Moreover, a significantly enhanced number of dead worms were observed after doxycycline.

***Conclusions.*** Targeting the *Wolbachia* in *O. volvulus* is effective in clearing microfilariae in the skin of onchocerciasis patients with persistent microfilaridermia and in enhanced killing of adult worms after repeated standard IVM treatment. Strategies can now be developed that include doxycycline to control onchocerciasis in areas where infections persist despite the frequent use of IVM.

***Clinical Trials Registration.*** ISRCTN 66649839.

## Abstract (precis)

Sub-optimal responses to ivermectin (IVM) have emerged. Targeting the *Onchocerca volvulus Wolbachia* endosymbionts with doxycycline is effective in clearing microfilariae in onchocerciasis patients with persistent microfilaridermia and in enhanced killing of adult worms after repeated standard IVM treatment.

Onchocerciasis affects >37 million people and remains a significant public health burden in developing countries [1, 2]. This infection is caused by the filarial nematode *Onchocerca volvulus*, residing in subcutaneous nodules where they produce millions of microfilariae (Mf) during their average life span of 10 years [3, 4]. The Mf migrate through the skin and eyes and are responsible for disease, such as dermatitis and visual impairment.

The African Programme for Onchocerciasis Control (APOC) relies on ivermectin (IVM) mass drug administration (MDA). However, the use of a single drug, of which more than a billion doses could potentially be needed for disease elimination [5], has raised concerns that adaptation of the parasites or the hosts [6], a reduction in effectiveness of the drug, or even drug resistance might occur and impair the success of the control programs. Resistance has occurred in several veterinary parasitic nematodes [7], and treatment failures have been suspected in other human helminthic infections [8–10].

In 2004, suboptimal response (SOR) to IVM was reported in onchocerciasis-endemic populations in communities of the Brong-Ahafo Region of Ghana [11, 12] with significant microfilaridermia despite a history of multiple (up to 14) MDAs with IVM. This indicates a considerable risk in continuing to rely on only increased IVM interventions [13]. A better intervention could be a chemotherapy leading to adulticidal or long-term sterilizing effects on the worms, at best with an already registered, repurposed drug.

Recently, our group has developed macrofilaricidal and long-term-sterilizing regimens based on the depletion of *Wolbachia* endosymbionts from *O. volvulus*. We demonstrated that doxycycline given at 100 mg/day for 5 or 6 weeks leads to a complete and irreversible (>2 years) sterilization of adult female worms. There is also a macrofilaricidal effect that can be first observed with 100 mg/day treatment by 5 weeks [14] and that seems to be more pronounced when doxycycline is administered at 200 mg/day [15].

Doxycycline is an antifilarial treatment that is more effective than the standard MDA regimen and is available to treat individual patients [16]. To formally prove the suitability of doxycycline in areas where MDA with IVM shows suboptimal performance, we carried out a placebo-controlled, double-blind, randomized study with 100 mg/day doxycycline administered for 6 weeks to patients with onchocerciasis in an area with multiple documented rounds of IVM treatment.

## METHODS

### Study Population and Area, Subject Eligibility, and Selection

The study was undertaken in 13 onchocerciasis-endemic communities located along the Pru, Lower Black Volta, and Tain river basins in the Brong-Ahafo Region of Ghana and includes communities where IVM SOR was reported [17, 18]. This study was approved by the Committee on Human Research, Publications and Ethics of the School of Medical Sciences of the Kwame Nkrumah University of Science and Technology, Kumasi, Ghana, and is registered at Current Controlled Trials (www.isrctn.com, identifier 66649839).

Volunteers were screened using the Onchocerciasis Control Programme registration books in combination with questionnaires to select those having microfilariae in the skin despite multiple previous treatments, and/or reappearance of palpable nodules [12]. Eligible volunteers had taken on average 10 rounds of IVM and 86% of the study participants had received ≥7 rounds (Table 1). Eligible participants were nodule carriers aged 18–50 years, >40 kg body weight, in good health, and without clinical conditions requiring chronic medication. Hepatic and renal functions as well as pregnancy tests were assessed by dipstick chemistry using venous blood and urine. Skin snips (biopsies) were taken to determine skin microfilarial loads. Exclusion criteria were abnormal hepatic and renal enzymes, pregnancy, breastfeeding, intolerance to doxycycline, alcohol or drug abuse, history of tuberculosis, glucose in urine, hypertension, or history of any condition requiring long-term medication (see also ISRCTN registry).

**Table 1.** Patient Baseline Data

Characteristic	Treatment		
	Doxycycline	Placebo	*P* Value
No. of patients	84	83	
Age, y			*P* = .004^a^
Mean ± SD	35.45 ± 9.46	39.60 ± 9.08	
Weight, kg			*P* = .079^a^
Mean ± SD	59.44 ± 8.25	57.23 ± 7.89	
Sex, No.			*P* = .289^b^
Female	18 (21.4%)	24 (28.9%)	
Male	66 (78.6%)	59 (71.1%)	
IVM rounds			*P* = .461^c^
Mean ± SD	10.40 ± 3.52	10.72 ± 3.36	
Min–Max	4–17	0–17	
Median	10	10	
25th, 75th percentiles	7.0, 13.8	9.0, 13.0	
No. of rounds			
0	0 (0%)	1 (1.2%)	
4	3 (3.6%)	2 (2.4%)	
5	3 (3.6%)	3 (3.6%)	
6	8 (9.5%)	4 (4.8%)	
7	8 (9.5%)	3 (3.6%)	
8	3 (3.6%)	3 (3.6%)	
9	10 (11.9%)	8 (9.6%)	
10	9 (10.7%)	18 (21.7%)	
11	9 (10.7%)	11 (13.3%)	
12	8 (9.5%)	7 (8.4%)	
13	2 (2.4%)	3 (3.6%)	
14	7 (8.3%)	7 (8.4%)	
15	8 (9.5%)	8 (9.6%)	
16	2 (2.4%)	1 (1.2%)	
17	4 (4.8%)	4 (4.8%)	
No. of palpated nodules			*P* = .279^c^
Total No.	206	198	
Mean ± SD	2.45 ± 1.41	2.39 ± 1.79	
Median	2	2	
No. of palpated sites			*P* = .339^c^
Total No.	136	127	
Mean ± SD	1.62 ± 0.79	1.53 ± 0.80	
Median	1	1	

Abbreviations: IVM, ivermectin; SD, standard deviation.

^a^ Student *t* test.

^b^ Fisher exact test.

^c^ Mann–Whitney *U* test.

### Study Design, Randomization, and Interventions

The primary objective of this randomized, placebo-controlled, double-blind trial was to assess the macrofilaricidal or sterilizing effect of doxycycline on adult female *O. volvulus* worms. Sample size was calculated based on the proportion of dead female *O. volvulus* worms observed in a previous study on doxycycline treatment [15]. Assuming a rate of 30% of patients with dead female worms in the placebo group and a rate of 62% in the doxycycline group, Fisher exact test (2-sided) at the level of α = 5% had a power of at least 90% with a sample size of 55 patients per study group. Allowing a dropout rate of 33%, the total number of patients per treatment arm was 83 individuals. To assure an equal distribution of Mf-positive and Mf-negative individuals to the treatment groups, eligible patients were randomized separately according to their Mf status by computer-generated random allocation sequence.

Participants received 100 mg doxycycline (Doxydoc) or matching placebo per day for 6 weeks under daily observation. Participants were instructed to continue participating in the ongoing MDA at 3 and 13 months after treatment. After study completion, doxycycline was offered to all placebo group participants and the Mf-positive patients from the doxycycline group.

### Nodule Mapping

The palpable nodules were mapped at study entry and at 20 months immediately prior to operation; the locations were assigned to a standardized system (head, shoulder, thorax, iliac crest, trochanter, os sacrum, knees). At 20 months, all palpable nodules were removed from the available patients.

### Laboratory Procedures

Skin Mf were assessed before and at 12 and 20 months after treatment onset. Two skin biopsies of 1–3 mg were taken from the buttocks and prepared for microscopic examination, as described previously [15].

Nodules were fixed in 80% ethanol and embedded in paraffin, and sections were stained with hematoxylin and eosin or Gomori method for iron, or immunostained against *Dirofilaria immitis Wolbachia* surface protein (Diwsp) and cathepsin D–like lysosomal aspartic protease of *O. volvulus* [15]. At least 8 sections across the nodules were histologically assessed. Worms that had been acquired after treatment according to the definition described previously [4] were subtracted from all analyses. Worms were classified as newly acquired, young, middle-aged, or old [19]. Sections were assessed independently by 2 experienced parasitologists (S. S., C. D. M.).

### Statistical Methods

Differences between the 2 treatment groups were analyzed with Student *t* test for quantitative parametric and the Mann–Whitney *U* test for all other nonparametric variables. Fisher exact test was used to compare binary qualitative variables and the Spearman rank correlation for comparisons of 2 quantitative parameters. Quantitative baseline values were compared to follow-up values using the sign test. Histological results were tested on single worm and on patient levels using the Mann–Whitney *U* test for quantitative and Fisher exact test for qualitative data. In addition, regression analyses were performed using alternating logistic regression, as implemented in the SAS procedure Genmod. This procedure allows testing for a potential dependency between the worms in 1 patient. Four data sets (per protocol [PP]; intention to treat [ITT]; treatment-PP; treatment and IVM-PP) were used to analyze the data and are explained in the relevant tables. Analyses were done using SPSS (IBM SPSS Statistics 22; Armonk, New York) and SAS version 9.2 (SAS Institute Inc, Cary, North Carolina).

## RESULTS

### Participant Flow and Adverse Reactions

The trial profile of the study is illustrated in Supplementary Figure 1. In all, 167 volunteers were enrolled and subsequently randomized into the treatment arms. Baseline data are given in Table 1. Importantly, 86% of study participants had taken ≥7 rounds of IVM (average 10 rounds). Statistical analysis showed that not only the Mf positivity (*P* = .86; ITT) but also the intensity of microfilaridermia (Mf/mg skin; *P* = .35; ITT) was independent of the IVM intake, consistent with the finding of suboptimal responses to IVM. Adherence to treatment was generally high. Doxycycline was well tolerated, with only mild adverse reactions (ARs) encountered (Supplementary Table 1). Twenty individuals (13 doxycycline, 7 placebo) did not complete the treatment course. Eleven patients (7 doxycycline, 4 placebo) dropped out because they reported ARs, such as diarrhea and vomiting, that the code-blinded medical doctors could not exclude from being potentially related to drug intake; all the other reasons for incompletion were reported as not drug-related. There was no significant difference in the number of ARs or in the types of ARs between the 2 treatment groups, except for nausea (grade 1), a common AR due to doxycycline intake.

### Sustained Reduction of Microfilarial Skin Loads in the Doxycycline Group

At baseline, there was no significant difference in the microfilarial load (median Mf/mg skin) between treatment groups (Table 2, all cohorts). However, at 12 months, a significant difference appeared (*P* < .001), with the doxycycline group showing medians of 0, whereas the placebo group's medians increased throughout all cohorts. This difference was maintained at 20 months after treatment (*P* < .001).

**Table 2.** Microfilarial Densities^a^

Study Time Point	ITT (n = 167)		Treatment-PP (n = 147)		Treatment and-IVM-PP (n = 87)	
	Doxycycline	Placebo	Doxycycline	Placebo	Doxycycline	Placebo
Baseline						
Mf positive	47/84 (56.0%)	48/83 (57.8%)	40/71 (56.3%)	43/76 (56.6%)	20/42 (47.6%)	23/45 (51.1%)
*P* value^b^	*P* = .876		*P* = 1.0		*P* = .831	
Mean ± SD	2.30 ± 6.29	3.23 ± 8.69	1.90 ± 4.35	3.46 ± 9.05	0.84 ± 2.66	3.27 ± 10.51
GM^c^	0.78	1.03	0.75	1.08	0.4	0.82
Min–Max	0–45.62	0–65.99	0–24.87	0–65.99	0–17.02	0–65.99
Median	0.15	0.26	0.14	0.27	0	0.13
25th, 75th percentiles	0, 1.11	0, 1.94	0, 1.11	0, 2.00	0, 0.63	0, 0.97
*P* value^d^	*P* = .495		*P* = .544		*P* = .498	
12 mo						
Mf positive	8/73 (11.0%)	44/72 (61.1%)	7/69 (10.1%)	42/70 (60.0%)	1/40 (2.5%)	24/44 (54.5%)
*P* value^b^	*P* < .001		*P* < .001		*P* < .001	
Mean ± SD	0.87 ± 3.74	4.50 ± 8.11	0.90 ± 3.85	4.33 ± 8.10	0.70 ± 4.43	4.01 ± 7.24
GM^c^	0.21	1.68	0.21	1.58	0.09	1.47
Min–Max	0–28.00	0–38.63	0–28.00	0–38.63	0–28.00	0–33.86
Median	0	0.96	0	0.81	0	0.52
25th, 75th percentiles	0, 0	0, 4.50	0, 0	0, 4.11	0, 0	0, 5.71
*P* value^d^	*P* < .001		*P* < .001		*P* < .001	
20 mo						
Mf positive	5/76 (6.6%)	50/74 (67.6%)	2/67 (3.0%)	47/70 (67.1%)	1/42 (2.4%)	27/45 (60.0%)
*P* value^b^	*P* < .001		*P* < .001		*P* < .001	
Mean ± SD	0.75 ± 4.57	3.99 ± 9.05	0.56 ± 4.35	3.88 ± 9.12	0.85 ± 5.49	1.87 ± 4.24
GM^c^	0.12	1.35	0.07	1.29	0.09	0.83
Min–Max	0–35.60	0–46.58	0–35.60	0–46.58	0–35.60	0–24.46
Median	0	0.65	0	0.52	0	0.21
25th, 75th percentiles	0, 0	0, 2.91	0, 0	0, 2.41	0, 0	0, 1.24
*P* value^d^	*P* < .001		*P* < .001		*P* < .001	
Persistently Mf positive^e^	1/69 (1.4%)	24/69 (34.8%)	0/65 (0%)	24/68 (35.3%)	0/40 (0%)	15/44 (34.1%)

Abbreviations: GM, geometric mean; ITT, intention to treat; IVM, ivermectin; Mf, microfilariae; PP, per protocol; SD, standard deviation.

^a^ The ITT set includes all patients who received the study drugs for at least 1 day. The Treatment-PP set includes all people who took the treatment according to protocol and on this basis another set was created, only including people who also took part in both rounds of mass drug administration between treatment and 20 months of follow-up (Treatment and IVM-PP).

^b^ Fisher exact test.

^c^ The GM was calculated by adding 1 to all values and after the calculation 1 was again subtracted from the result.

^d^ Mann–Whitney *U* test.

^e^ Number of participants who were persistently Mf positive over all 3 timepoints.

Of the 87 study participants who completed the treatment PP and received 2 rounds of IVM during the course of the trial (treatment and IVM-PP), 49.4% were microfilaridermic (Mf positive) at study entry. At the 12-month follow-up time point, only 1 of 40 individuals (2.5%) in the doxycycline treatment group remained microfilaridermic, indicating a significant drop after treatment. In contrast, 24 of 44 patients (54.5%) in the placebo group remained Mf-positive despite 1 additional round of IVM (*P* < .001). Importantly, even after 2 additional rounds of IVM at the 20-month follow-up, 34.1% of individuals in the placebo group were persistently Mf positive.

In the ITT dataset, a few more patients in the doxycycline treatment arm remained microfilaridermic, consistent with the fact that 13 patients did not complete the doxycycline regimen. Interestingly, in the PP dataset, 7 of 69 patients were still Mf positive after 12 months, whereas this number dropped to 2 of 67 patients after 20 months, consistent with a block in new embryo production and therefore slow decay of Mf in the absence of IVM.

In summary, the Mf data reflect the long-term sterilization of *O. volvulus* after treatment with doxycycline, whereas fertility of female worms was not reduced in placebo patients. The results from the placebo group are of particular concern, as despite 2 additional rounds of IVM, Mf positivity still remained prevalent in this group.

### Doxycycline Inhibits Embryogenesis and Has Macrofilaricidal Activity

All the histological analyses were performed according to PP analysis—that is, included only those individuals who had completed doxycycline/placebo treatment and adhered to the 20-month follow-up. Worms were classified as having many, few, or no *Wolbachia* (Table 3), as described previously [15]. Eighty percent of the female worms in the placebo group were *Wolbachia* positive (few and many), whereas only 5.1% contained *Wolbachia* in the doxycycline-treated group (*P* < .001). A similar difference was observed for male worms.

**Table 3.** Effect of Doxycycline Treatment on Presence of *Wolbachia* in Worms

Treatment Group	Time, mo	No. of Patients/ Nodules^a^	No. of Living Female Worms^b^				No. of Living Male Worms^b^			
			All^c^	With *Wolbachia*^d^			All^c^	With *Wolbachia*^d^		
				Many	Few	None		Many	Few	None
Doxycycline (6 wk)	20	56/152	117	1	5	111	41	0	3	36
Placebo	20	63/199	184	60	80	35	54	5	19	26

^a^ Only evaluable patients/nodules are included; nodules with newly acquired worms only are subtracted.

^b^ Newly acquired worms were subtracted (doxycycline, n = 8 females; placebo, n = 7 females).

^c^ Although the number of all living worms is given to make the numbers consistent to the other tables, in 15 worms, it was not possible to distinguish if the worm had many, few, or no *Wolbachia*, due to too little worm material in the respective histological sections. Therefore, “All” is not always a summary of the 3 categories.

^d^ Significant difference between the doxycycline and the placebo group regarding absence of *Wolbachia* (^b^*P* < .001 for female and male worms respectively, Fisher exact test).

When investigating for embryogenesis, we found that, in accordance to our earlier study [15], 53% of female worms in the placebo group had normally developed embryos at 20 months, and Mf were observed in 30.7% of the nodules analyzed (Table 4). In contrast, only 3.8% female worms from the doxycycline group exhibited normal embryogenesis, and none of the 152 nodules were Mf positive. In addition to the inhibition of embryogenesis, we also observed a significant reduction in the insemination of the female worms in this group (Table 4).

**Table 4.** Effect of Doxycycline Treatment on Embryogenesis

Treatment Group	Time, mo	No. of Patients/Nodules^a^	No. of Living Female Worms^b^						No. of Nodules	
			All	Embryos^c^				Sperms in Uterus^d^	All	With Intact Microfilariae^e^
				Not Judgeable	Uterus Empty, Oocytes Only	Normal	Degenerated			
Doxycycline (6 wk)	20	56/152	117	13	93	4	7	9	152	0
Placebo	20	63/199	184	16	76	89	3	54	199	61

^a^ Only evaluable patients/nodules are included; nodules with newly acquired worms only are subtracted.

^b^ Newly acquired worms were subtracted (doxycycline, n = 8 females; placebo, n = 7 females).

^c^ Significant difference between the doxycycline and the placebo group regarding the presence of normal vs degenerated embryogenesis (*P* < .001, Fisher exact test).

^d^ Significant difference between the doxycycline and the placebo group regarding the number of worms found with sperms within the uterus (*P* < .001, Fisher exact test).

^e^ Significant difference between the doxycycline and the placebo group regarding the proportion of nodules with intact microfilariae (*P* < .001, Fisher exact test).

The doxycycline group showed a significant increase in dead female worms at 20 months compared with the placebo group (Table 5; *P* < .001). A trend toward a higher percentage of dead males in the doxycycline-treated group was also seen (*P* = .136); the variability may be due to the observation that dead males are more quickly absorbed than females [20]. The age pattern of the worm population did not reveal significant differences between the treatment groups (Table 6).

**Table 5.** Macrofilaricidal Effect of Doxycycline Treatment

Treatment Group	Time, mo	No. of Patients/Nodules^a^	No. of Female Worms^b^		No. of Male Worms^b^	
			All	Dead Females^c^ (%)	All	Dead Males (%)
Doxycycline (6 wk)	20	56/152	253	136 (54%)	50	9 (18%)
Placebo	20	63/199	300	116 (39%)	58	4 (7%)

^a^ Only evaluable patients/nodules are included; nodules with newly acquired worms only were subtracted.

^b^ Newly acquired worms were subtracted (doxycycline, n = 8 females; placebo, n = 7 females).

^c^ Significant difference between the doxycycline and the placebo group regarding the proportion of dead female worms (*P* < .001, Fisher exact test).

**Table 6.** Age Pattern of Worms^a^

Treatment Group	Time, mo	No. of Patients/Nodules^b^	No. of Living Female Worms					No. of Living Male Worms				
			All	Age				All^c^	Age			
				Newly Acquired	Young	Middle	Old		Newly Acquired	Young	Middle	Old
Doxycycline (6 wk)	20	56/157	125	8	12	48	57	41	0	2	27	10
Placebo	20	63/205	191	7	30	73	81	55	1	2	34	17

^a^ Characteristics for the age of a worm included loss of body wall integrity, loss of nuclei of all organs, and absence of aspartic protease (APR) staining. Very degenerated worms, still APR positive, were classified as moribund and grouped in the category “dead.” Older worms were larger and presented degenerated tissues. Gomori iron stain showed that the worms accumulated more iron with increasing age, first in the gut and later in other organs. Using an antibody against *O. volvulus* lysosomal aspartic protease, the gut of young worms was stained only weakly, whereas it was stronger in older worms, accompanied by additional staining of hypodermis and epithelia. These criteria as well as morphological findings (diameter, position within the nodule) were used to discriminate newly acquired worms [4, 15].

^b^ Only evaluable patients/nodules are included. In contrast to Tables 4–6, newly acquired worms are included.

^c^ In 3 male worms, it was not possible to distinguish the age pattern of the worm in the respective histological sections. Therefore, “All” is not always a summary of the 4 categories.

Table 7 shows the average numbers of sites and nodules, palpated at study entry and at 20 months prior to nodulectomy, as well as the actually ectomized and evaluated nodules. There was a greater reduction in the number of palpated nodules in the doxycycline group over the observation period compared with the placebo group (*P* = .015). These data suggest that dead worms are absorbed over time and that the macrofilaricidal effect of doxycycline may even have been greater than detected by histology.

**Table 7.** Palpated, Operated, and Evaluated Nodules and Sites^a^

Study Time Point	No. of Nodules		No. of Sites	
	Doxycycline	Placebo	Doxycycline	Placebo
Baseline				
No. of patients	71	76	71	76
Total No. of nodules/sites	178	182	114	116
Mean ± SD	2.51 ± 1.40	2.39 ± 1.86	1.61 ± 0.78	1.53 ± 0.81
Min–Max	1–6	1–11	1–4	1–4
Median	2	2	1	1
25th, 75th percentiles	1, 3	1, 3	1, 2	1, 2
*P* value^b^	*P* = .151		*P* = .378	
20 mo				
No. of patients	67	69	67	69
Total No. of nodules/sites	137	150	98	104
Mean ± SD	2.04 ± 1.32	2.17 ± 1.68	1.46 ± 0.68	1.51 ± 0.87
Min–Max	1–6	0–8	1–4	0–4
Median	2	2	1	1
25th, 75th percentiles	1, 3	1, 2	1, 2	1, 2
*P* value^b^	*P* = .858		*P* = .903	
Difference 20 mo – baseline				
No. of patients	67	69	67	69
Mean ± SD	−0.43 ± 1.03	−0.09 ± 1.00	−0.12 ± 0.54	0.01 ± 0.56
Min–Max	−5 to 2	−4 to 2	−1 to 1	−2 to 1
Median	0	0	0	0
25th, 75th percentiles	−1, 0	0, 0	0, 0	0, 0
*P* value^b^	*P* = .015		*P* = .109	
Operated^c^				
No. of patients	66	66	66	66
Total No. of nodules/sites	182	227	99	108
Mean ± SD	2.76 ± 2.18	3.44 ± 2.79	1.5 ± 0.75	1.64 ± 0.97
Min–Max	1–13	1–14	1–4	1–5
Median	2	2.5	1	1
25th, 75th percentiles	1, 3	2, 4	1, 2	1, 2
*P* value^b^	*P* = .126		*P* = .591	
Evaluated^d^				
No. of patients	56	63	56	63
Total No. of nodules/sites	152	199	79	97
Mean ± SD	2.71 ± 2.02	3.16 ± 2.39	1.41 ± 0.73	1.54 ± 0.84
Min–Max	1–11	1–12	1–4	1–4
Median	2	2	1	1
25th, 75th percentiles	1, 3	2, 4	1, 2	1, 2
*P* value^b^	*P* = .277		*P* = .412	

Abbreviation: SD, standard deviation.

^a^ The set used here (Treatment-per protocol) includes all people who took the treatment (doxycycline or placebo) according to protocol.

^b^ Mann–Whitney *U* test.

^c^ In 4 participants, no nodule was found before or after incision.

^d^ Thirteen participants and, in total, 11.5% (n = 47) of all nodules could not be analyzed for parasitic status; the reasons for this exclusion included extensive calcification of the nodule, or being of nononchocercal origin (eg, foreign body granulomas, lipomas, or lymph nodes). An additional 11 nodules were subtracted for containing newly acquired worms only.

### Additional Statistical Analyses

In addition to the PP analysis, we reanalyzed the data using the ITT data set. Table 8 shows in summary that even under ITT conditions, all parameters compared were still significant, this being the case at the level of the worm itself as well as with the total sum of the worms present per patient. Details on the ITT dataset for presence of *Wolbachia*, embryogenesis, and worm killing can be found in Supplementary Tables 2–5.

**Table 8.** Additional Statistical Analysis^a^

	Per Patient^b^		Per Single Worm^c^		Regression Analysis^d^ (Repeated Factor: Patient)	
	PP	ITT	PP	ITT	PP	ITT
Dead females	*P* = .097	*P* = .091	*P* < .001	*P* = .001	*P* = .006	*P* = .011
			OR = 1.8438	OR = 1.6987	OR = 1.9809	OR = 1.8134
Live females	*P* = .028	*P* = .054	*P* < .001	*P* = .001	*P* = .006	*P* = .011
			OR = 0.5424	OR = 0.5887	OR = 0.5048	OR = 0.5514
Many bacteria (vs no bacteria)	*P* < .001	*P* < .001	*P* < .001	*P* < .001	*P* < .001	*P* < .001
			OR = 0.0053	OR = 0.0609	OR = 0.0056	OR = 0.0949
Few bacteria (vs no bacteria)	*P* < .001	*P* < .001	*P* < .001	*P* < .001	*P* < .001	*P* < .001
			OR = 0.0197	OR = 0.0444	OR = 0.019	OR = 0.044
No bacteria (vs few ± many bacteria)	*P* < .001	*P* < .001	*P* < .001	*P* < .001	*P* < .001	*P* < .001
			OR = 74	OR = 19.2287	OR = 67.3149	OR = 15.561
Normal vs degenerated embryogenesis	*P* < .001	*P* < .001	*P* < .001	*P* < .001	*P* < .001	*P* < .001
			OR = 0.0193	OR = 0.0666	OR = 0.0234	OR = 0.0844
Sperms in uterus	*P* < .001	n.d.^e^	*P* < .001	n.d.^e^	*P* < .001	n.d.^e^
			OR = 0.2006		OR = 0.1337	

Abbreviations: ITT, intention to treat; n.d., not done; OR, odds ratio; PP, per protocol.

^a^ The PP data set included all participants who took the treatment according to protocol and were present for the nodulectomies at 20 months. This data set was used for the primary analyses of all histological data. The ITT set included all patients who received the study drugs for at least 1 day. This data set was used to describe all baseline data and the adverse reactions. It was additionally used to confirm the primary PP analysis. In this case, missing values from participants who were not present for nodulectomies (n = 19) were replaced by a worst-case scenario (1 nodule containing 1 live female, *Wolbachia*-positive worm with normal embryogenesis and intact microfilariae).

^b^ Analysis was performed on the per-patient level using the Mann–Whitney *U* test.

^c^ Analysis was performed on the single-worm level using Fisher exact test.

^d^ Analysis was performed by regression analysis (Proc Genmod) with the patient as repeated factor to account for dependence within patient. ORs are given for the doxycycline group.

^e^ Not done as the ITT worst-case scenario was not defined for this parameter.

Because the worms within one patient could not, a priori, be regarded as independent (for example, they aggregate in nodules and this may facilitate their survival, and doxycycline may have had different bioavailability in different patients), we used alternating logistic regression to account for within-patient dependence (Table 8); despite the additional factor, results remained significant.

It was observed that the mean age differed between the treatment groups, which randomly occurred during the randomization process. Although multiple regression analysis did not show any influence of the patient's age on most of the histological outcome variables, it revealed different age effects in the treatment groups regarding the occurrence of dead worms (Supplementary Figure 2). A higher age in the placebo group was associated with an increased likelihood for dead worms (odds ratio [OR], 1.0771; *P* = .005), but this age effect was no longer present in the doxycycline group (OR, 1.0092; *P* = .5775).

## DISCUSSION

There is now a consensus that chemotherapies beyond monotherapy with IVM are urgently needed for onchocerciasis. A number of situations and challenges underscore this reality: (1) individual cases presenting to doctors need to be given the best available treatment; (2) in *Loa loa–*coendemic areas, patients coinfected with loiasis with *Loa* Mf loads >8000 Mf/mL run a risk of developing serious, even fatal, neurologic reactions when treated with IVM as part of onchocerciasis MDA [21, 22]; (3) new agents are also needed should IVM resistance, or indeed any other form of SOR, occur.

The use of doxycycline fulfills these criteria for situations 1 and 2 for onchocerciasis. In situation 1, doxycycline's macrofilaricidal and long-term sterilizing activities render it a disease-curing choice for individual treatment, different to IVM, which will have to be repeated for years until the adult worms have terminally stopped embryogenesis [16]. The treatment recommendation here has been to administer 1 course of doxycycline followed by 1 round of IVM, to combine the sterilizing and killing effects of doxycycline on the female worms with the rapid killing of skin Mf by IVM [23].

In situation 2, recent consensus is to identify areas with a likelihood for coinfected individuals with high counts for *L. loa* Mf, and then to test (eg, with cell phone–based microscopy) all individuals in such areas and exclude those with >30 000 Mf/mL from IVM treatment [24]. However, those individuals can be treated safely with doxycycline to kill their *O. volvulus* parasites, as *L. loa* does not contain *Wolbachia*. In their 2013 annual meeting in Brazzaville, the Joint Action Forum, APOC's governing board, has adopted the use of doxycycline for treatment of individual patients (http://www.who.int/apoc/Journal_ENGLISH_Day_3_FINAL_red.pdf).

The present study delivers formal proof that doxycycline, given at 100 mg/day for 6 weeks, is also suitable for situation 3, in areas with SOR. This dose interrupted embryogenesis for at least 20 months, whereas the placebo group did not clear skin Mf even after receiving 2 additional doses of IVM; this latter group is typical of the SOR to IVM observed previously [11, 12, 17, 18]. Previous investigations in our study area have suggested that the presence of fertile female worms 3–6 months after the last of at least 9 rounds of IVM is inconsistent with a normal response to IVM [11, 12, 17, 18]. The results from the placebo group in our study underscore this further, while the doxycycline regimen delivers a safe “ready-to-go” approach for *O. volvulus* worms suspected of showing SOR to IVM. Community trials in our study area are currently addressing the impact of a test-and-treat strategy, using doxycycline in communities where SOR to IVM has been observed.

Doxycycline did not provoke a different level of ARs compared with the placebo group.

As opposed to the only known macrofilaricide suramin [25], the low toxicity of doxycycline is probably due to its relatively slow-acting effect on the worms, leading to a protracted death after more than a year [15, 26], ensuring that the host is able to handle the events occurring during the killing of the adults; as a consequence, severe ARs do not occur. The slow macrofilaricidal action of the drug, however, brings about an issue regarding monitoring in clinical trials if they take place in endemic areas. Although a 100% macrofilaricidal activity of doxycycline would probably be achieved, as model estimates go [27], this cannot be directly observed in trials because with observation periods >1.5 years, there is confounding interference from new incoming worms in geographical areas such as our study site, where infection transmission has not yet been interrupted [4, 15].

In the present trial, the proportion of dead worms analyzed in the available nodules after doxycycline was 54% (PP). A daily dose of “only” 100 mg/day as opposed to 200 mg/day [15] probably does not account for a lower number of dead worms, as new modeling data suggest that regardless of the daily dose, the average life span of a worm is reduced from the normal 10 years' average to 2 years after exposure to doxycycline for 4–6 weeks [28].

In addition, the actual macrofilaricidal rate was probably higher as we observed a loss of nodules between pretreatment and 20 months in the doxycycline group. Taking into account that about 0.4 nodules per patient were lost in this group (Table 7), with an average of about 1.5 female worms per nodule (Table 5), one would have to include approximately 30 more dead female worms to the death toll, raising the ratio to approximately 59%. Interestingly, it has been observed that the size of *Onchocerca ochengi* nodules is significantly reduced after tetracycline treatment in cattle and indicates absorption of the nodules [29].

The ultimate cause of the SOR to IVM is as yet unknown. Although selection of resistance genes has been discussed [30–33], and evidence of allele selection could indeed be documented from worms of our study area, there are other potential mechanisms, such as adaptation of the worms' biology (eg, change in transcription levels as opposed to gene mutation), or even immunological facilitation by the host. Along these lines, we have observed that the level of TGF-β detectable in host cells around female worms in nodules are associated with the rounds of IVM [34]. If these preliminary data hold for other areas, this type of SOR may become much more frequently observed and will require adequate therapeutic approaches, such as killing adult worms or sterilizing them permanently. This study shows that doxycycline treatment fulfills these requirements.

## Supplementary Data

Supplementary materials are available at *Clinical Infectious Diseases* online (http://cid.oxfordjournals.org). Supplementary materials consist of data provided by the author that are published to benefit the reader. The posted materials are not copyedited. The contents of all supplementary data are the sole responsibility of the authors. Questions or messages regarding errors should be addressed to the author.

Supplementary Data
