# Supplementary material for: Doxycycline Leads to Sterility and Enhanced Killing of Female Onchocerca volvulus Worms in an Area With Persistent Microfilaridermia After Repeated Ivermectin Treatment: A Randomized, Placebo-Controlled, Double-Blind Trial
Source: Clin Infect Dis. 2015 May 6;61(4):517–26. doi: 10.1093/cid/civ363 (PMC4518165; doi:10.1093/cid/civ363)
Supplement: Supplementary Data [file supp_61_4_517__index.html]

Doxycycline leads to sterility and enhanced killing of female Onchocerca volvulus worms in an area with persistent microfilaridermia after repeated ivermectin treatment – a randomized placebo controlled double-blind trial — Doxycycline Leads to Sterility and Enhanced Killing of Female Onchocerca volvulus Worms in an Area With Persistent Microfilaridermia After Repeated Ivermectin Treatment: A Randomized, Placebo-Controlled, Double-Blind Trial — Doxycycline Leads to Sterility and Enhanced Killing of Female Onchocerca volvulus Worms in an Area With Persistent Microfilaridermia After Repeated Ivermectin Treatment: A Randomized, Placebo-Controlled, Double-Blind Trial — Supplementary Data 

# Doxycycline Leads to Sterility and Enhanced Killing of Female *Onchocerca volvulus* Worms in an Area With Persistent Microfilaridermia After Repeated Ivermectin Treatment: A Randomized, Placebo-Controlled, Double-Blind Trial

## Supplementary Data

Supplementary Data

- Supplementary Tables - docx file
- Supplementary Figures - pdf file
