# Supplementary material for: Doxycycline Leads to Sterility and Enhanced Killing of Female Onchocerca volvulus Worms in an Area With Persistent Microfilaridermia After Repeated Ivermectin Treatment: A Randomized, Placebo-Controlled, Double-Blind Trial
Source: Clin Infect Dis. 2015 May 6;61(4):517–26. doi: 10.1093/cid/civ363 (PMC4518165; doi:10.1093/cid/civ363)
Supplement: Supplementary Data [file supp_civ363_civ363supp_tables.docx]

Supplementary Table 1: Adverse reactions

|  | | Treatment | |
| --- | --- | --- | --- |
|  |  | Doxycycline | Placebo |
| No. of participants with AR | | 48/84 (57.1%) | 59/83 (71.1%) |
|  | | *p* = 0.076^a^ | |
| No. of different AR per affected participants | Mean + SD | 1.67+1.00 | 1.68+0.99 |
|  | Min - Max | 1 - 6 | 1 - 6 |
|  | Median | 1 | 1 |
|  | 25th;75th percentiles | 1.0; 2.0 | 1.0; 2.0 |
|  |  | *p* = 0.903^b^ | |
|  | 1 | 27/48 (56.3%) | 32/59 (54.2%) |
|  | 2 | 14/48 (29.2%) | 19/59 (32.2%) |
|  | 3 | 5/48 (10.4%) | 6/59 (10.2%) |
|  | 4 | 1/48 (2.1%) | 0/59 (0.0%) |
|  | 5 | 0/48 (0%) | 1/59 (1.7%) |
|  | 6 | 1/48 (2.1%) | 1/59 (1.7%) |
|  | Headache | 12 | 15 |
|  | Itching | 9 | 18 |
|  | Fever | 2 | 5 |
|  | Stomach pain | 7 | 8 |
|  | Diarrhoea | 8 | 8 |
|  | Bloody stool | 1 | 3 |
|  | Bloody diarrhoea^c^ | 2 | 0 |
|  | Nausea^d^ | 7 | 0 |
|  | Vomiting | 2 | 1 |
|  | Dizziness | 2 | 1 |
|  | Body pain | 2 | 7 |
|  | Chest pain | 1 | 4 |
|  | Waist pain | 8 | 12 |
|  | Joint pain | 5 | 4 |
|  | Malaria | 3 | 1 |
|  | Others | 9 | 12 |

SD = Standard deviation

^a^ Fisher´s exact test

^b^ Mann-Whitney U-test

^c^ Although the bloody diarrhoea was not serious at any time point, treatment was stopped preemptively.

^d^ Nausea (Grade 1) was the only AE with a significant difference between doxycycline and placebo treated participants (p = 0.014, Fisher’s exact test)

Supplementary Table 2:

Effect of doxycycline treatment on presence of *Wolbachia* in worms

| Treatment group | Time  (months) | Number of patients^a^/ nodules^b^ | Number of living female worms | | | | Number of living male worms | | | |
| --- | --- | --- | --- | --- | --- | --- | --- | --- | --- | --- |
|  |  |  | All^c^ | With *Wolbachia*^d^ | | | All^c^ | With *Wolbachia*^d^ | | |
|  |  |  |  | Many | Few | None |  | Many | Few | None |
| Doxycycline  (6 weeks) | 20 | 62/173  (71/182) | 135  (144) | 5  (14) | 12  (12) | 118  (118) | 45 | 1 | 4 | 38 |
| Placebo | 20 | 66/219  (76/229) | 198  (208) | 64  (74) | 87  (87) | 38  (38) | 64 | 6 | 25 | 29 |

^a^ 19 participants (Doxycycline N = 9, Placebo N = 10) did not show up for nodulectomies. Missing values for these 19 participants were replaced by by a worst case scenario (1 nodule containing 1 live female, *Wolbachia*-positive worm with normal embryogenesis and intact mf). The numbers for the ITT analysis incl. the replaced missing values are shown in brackets.

In 5 participants (Doxycycline N = 1, Placebo N = 4) no nodule was found before or after incision and the nodules of another 15 participants (Doxycycline N = 12, Placebo N = 3) could not be analysed; the reasons being extensive calcification, non-onchocercal origin (e.g. foreign body granulomas, lipomas or lymph nodes).

^b^ The number of nodules is the sum of all evaluable nodules minus the number of nodules with newly acquired worms only (N = 11; Doxycycline N = 5, Placebo N = 6).

^c^ While the number of all living worms is given to make the numbers consistent to the other tables, in 15 worms (9 female, 6 male) it was not possible to distinguish if the worm had many, few or no *Wolbachia*, due to too little worm material in the respective histological sections. Therefore, “All” is not always a summary of the three categories.

^d^ Significant difference between the doxycycline and the placebo group regarding the absence of *Wolbachia* (^b^ *p* < 0.001 (p < 0.001) for female and male worms respectively, Fisher´s exact test).

Supplementary Table 3: Effect of doxycycline treatment on embryogenesis

| Treatment group | Time  (months) | Number of patients^a^/ nodules^b^ | Number of living female worms | | | | | | Number of nodules | |
| --- | --- | --- | --- | --- | --- | --- | --- | --- | --- | --- |
|  |  |  | All | Embryos^c^ | | | | Sperms  in uterus^d^ | All | With intact mf^e^ |
|  |  |  |  | not judgeable | uterus empty, oocytes only | normal | dege-nerated |  |  |  |
| Doxycycline  (6 weeks) | 20 | 62/173  (71/182) | 135  (144) | 15  (15) | 106  (106) | 7  (16) | 7  (7) | 9 | 173  (182) | 2  (11) |
| Placebo | 20 | 66/219  (76/229) | 198  (208) | 17  (17) | 85  (85) | 93  (103) | 3  (3) | 55 | 219  (229) | 64  (74) |

^a^ 19 participants (Doxycycline N = 9, Placebo N = 10) did not show up for nodulectomies. Missing values for these 19 participants were replaced by by a worst case scenario (1 nodule containing 1 live female, *Wolbachia*-positive worm with normal embryogenesis and intact mf). The numbers for the ITT analysis incl. the replaced missing values are shown in brackets.

In 5 participants (Doxycycline N = 1, Placebo N = 4) no nodule was found before or after incision and the nodules of another 15 participants (Doxycycline N = 12, Placebo N = 3) could not be analysed; the reasons being extensive calcification, non-onchocercal origin (e.g. foreign body granulomas, lipomas or lymph nodes).

^b^ The number of nodules is the sum of all evaluable nodules minus the number of nodules with newly acquired worms only (N = 11; Doxycycline N = 5, Placebo N = 6).

^c^ Significant difference between the doxycycline and the placebo group regarding the presence of normal vs. degenerated embryogenesis(*p* < 0.001 (p < 0.001), Fisher´s exact test).

^d^ Significant difference between the doxycycline and the placebo group regarding the number of worms found with sperms within the uterus (*p* < 0.001, Fisher´s exact test).

^e^ Significant difference between the doxycycline and the placebo group regarding the proportion of nodules with intact mf (*p* < 0.001 (p < 0.001), Fisher´s exact test).

Supplementary Table 4: Macrofilaricidal effect of doxycycline treatment

| Treatment group | Time  (months) | Number of patients^a^/ nodules^b^ | Number of female worms | | Number of male worms | |
| --- | --- | --- | --- | --- | --- | --- |
|  |  |  | All | Dead females / %^c^ | All | Dead males / % |
|  |  |  |  |  |  |  |
| Doxycycline  (6 weeks) | 20 | 62/173  (71/182) | 282  (291) | 147 / 52%  (147 / 51%) | 54 | 9 / 17% |
| Placebo | 20 | 66/219  (76/229) | 323  (333) | 125 / 39%  (125 / 37%) | 68 | 4 / 6% |

^a^ 19 participants (Doxycycline N = 9, Placebo N = 10) did not show up for nodulectomies. Missing values for these 19 participants were replaced by by a worst case scenario (1 nodule containing 1 live female, *Wolbachia*-positive worm with normal embryogenesis and intact mf). The numbers for the ITT analysis incl. the replaced missing values are shown in brackets.

In 5 participants (Doxycycline N = 1, Placebo N = 4) no nodule was found before or after incision and the nodules of another 15 participants (Doxycycline N = 12, Placebo N = 3) could not be analysed; the reasons being extensive calcification, non-onchocercal origin (e.g. foreign body granulomas, lipomas or lymph nodes).

^b^ The number of nodules is the sum of all evaluable nodules minus the number of nodules with newly acquired worms only (N = 11; Doxycycline N = 5, Placebo N = 6).

^c^ Significant difference between the doxycycline and the placebo group regarding the proportion of dead female worms (*p* = 0.001 (p = 0.001), Fisher´s exact test).

Supplementary Table 5: Age pattern of worms

| Treatment group | Time  (months) | Number of patients^a^/ nodules^b^ | Number of living female worms | | | | | Number of living male worms | | | | |
| --- | --- | --- | --- | --- | --- | --- | --- | --- | --- | --- | --- | --- |
|  |  |  | All | age | | | | All^c^ | age | | | |
|  |  |  |  | Newly acquired | young | middle | old |  | Newly acquired | young | middle | old |
| Doxycycline  (6 weeks) | 20 | 62/178 | 144 | 9 | 13 | 58 | 64 | 45 | 0 | 2 | 30 | 11 |
| Placebo | 20 | 66/225 | 205 | 7 | 32 | 76 | 90 | 65 | 1 | 4 | 39 | 20 |

^a^ 19 participants (Doxycycline N = 9, Placebo N = 10) did not show up for nodulectomies. A replacement of missing values was not planned for the age pattern of worms. Therefore only ITT-values without replacement are shown here.

In 5 participants (Doxycycline N = 1, Placebo N = 4) no nodule was found before or after incision and the nodules of another 15 participants (Doxycycline N = 12, Placebo N = 3) could not be analysed for parasitic status; the reasons for this exclusion included extensive calcification of the nodules, or being of non-onchocercal origin (e.g. foreign body granulomas, lipomas or lymph nodes).

^b^ In contrast to supplementary tables 3-5, the number of nodules incl. the nodules with newly acquired worms only are shown.

^c^ in 3 male worms it was not possible to distinguish the age pattern of the worm in the respective histological sections. Therefore, “All” is not always a summary of the four categories.
