# Supplementary material for: Doxycycline Leads to Sterility and Enhanced Killing of Female Onchocerca volvulus Worms in an Area With Persistent Microfilaridermia After Repeated Ivermectin Treatment: A Randomized, Placebo-Controlled, Double-Blind Trial
Source: Clin Infect Dis. 2015 May 6;61(4):517–26. doi: 10.1093/cid/civ363 (PMC4518165; doi:10.1093/cid/civ363)
Supplement: Supplementary Data [file supp_civ363_civ363supp_figs.pdf]

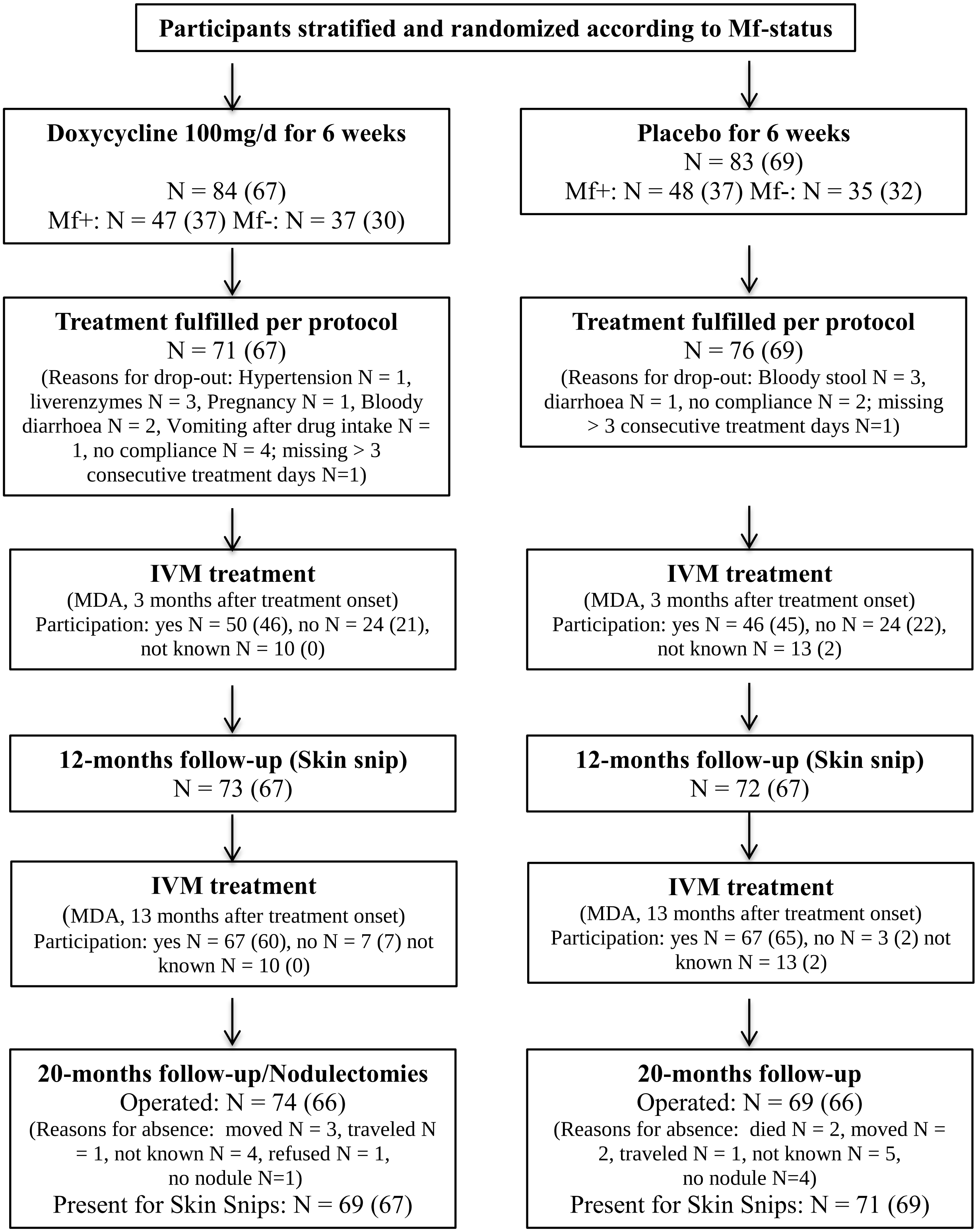

Supplementary Figure 1: Flow chart of volunteers who took part in the study.

The chart shows the number of individuals analysed according to the intention-to-treat (per protocol) principle. There was no significant difference in the proportions of non-attendance to the different follow-up time points between individuals randomized to the doxycycline and those randomized to the placebo group, neither with regard to the presence for nodulectomy nor for skin mf determination.

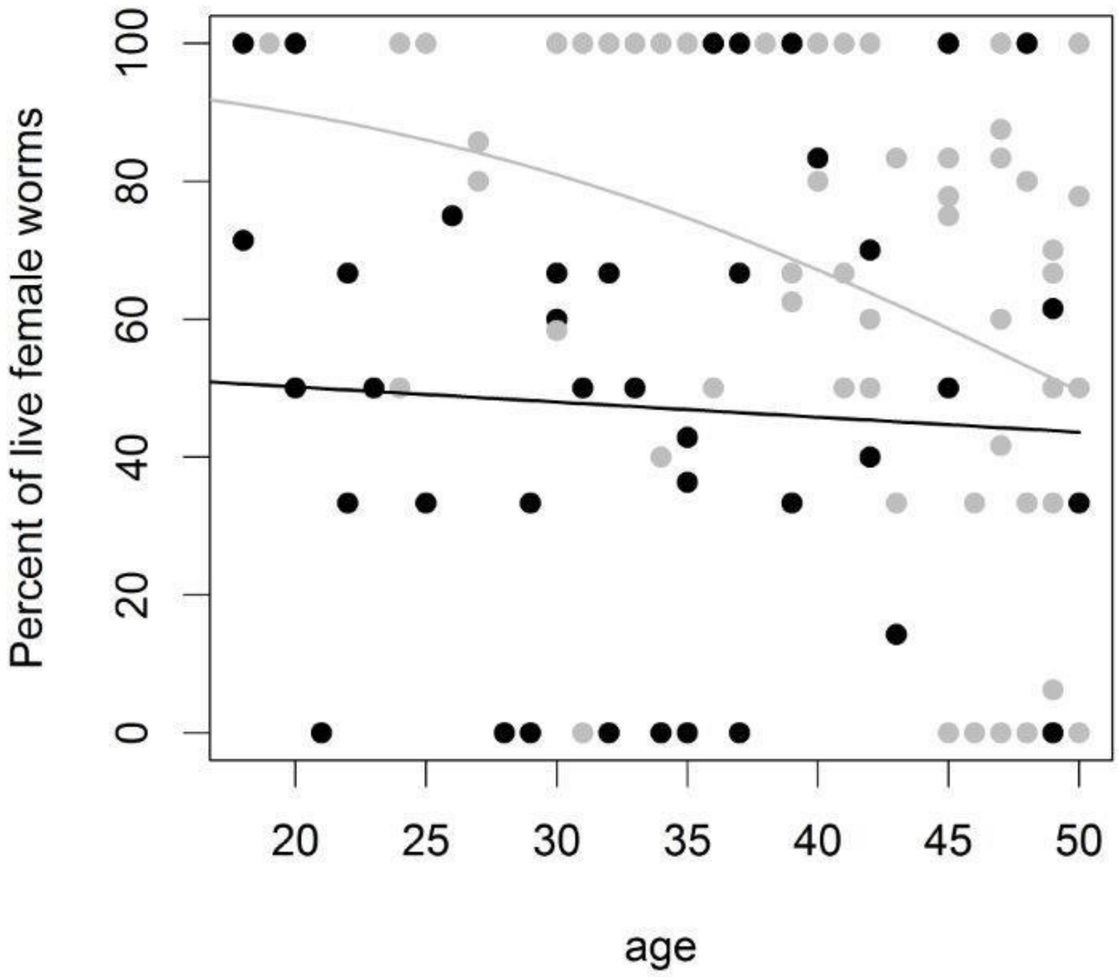

Supplementary Figure 2: Estimated effects of the covariates age of the individual and treatment on the presence of live worms. Using Proc Genmod procedure for logistic regression analysis shows that while in the placebo group (grey) the number of life worms decreases with age, this age effect is not present anymore in the doxycycline group (black) after the treatment.
